# Effects of different levels of Citri Sarcodactylis Fructus by-products fermented feed on growth performance, serum biochemical, and intestinal health of cyan-shank partridge birds

**DOI:** 10.1038/s41598-023-47303-5

**Published:** 2023-11-17

**Authors:** Xinhong Zhou, Huaidan Zhang, Shiyi Li, Yilong Jiang, Jicheng Deng, Chuanpeng Yang, Xianxin Chen, Li Jiang

**Affiliations:** 1 Leshan Academy of Agriculture Science, Leshan, 614001 Sichuan China; 2 College of Life Science and Engineering, Southwest University of Science and Technology, Mianyang, 621010 Sichuan China

**Keywords:** Biological techniques, Zoology

## Abstract

This research aimed to investigate the effects of supplements containing fermented feed made from Citri Sarcodactylis Fructus by-products (CSFBP-Fermented feed) on the growth performance, immunological function, and gut health of broilers. 1080 cyan-shank partridge birds aged 47 days were chosen and casually distributed to four groups, each with 6 replicates and 45 birds per replicate. The experimental groups were provided with 1% (group T2), 3% (group T3) and 5% (group T4) of CSFBP-fermented feed in the basic diet, while the control group (group T1) received the basic diet. The findings revealed that supplementation with CSFBP-Fermented feed reduced ADFI and FCR and improved ADG in birds (*P* < 0.05). MDA levels in the serum of birds fed CSFBP-fermented feed were lower than in the control group (*P* < 0.05). The CAT activity in the serum of broilers increased after supplementation with 3% CSFBP-Fermented feed (*P* < 0.05). Supplementing broilers with CSFBP-fermented feed enhanced VH in the ileum, jejunum, and duodenum (*P* < 0.05). The addition of 3% CSFBP-Fermented feed decreased CD in the jejunum (*P* < 0.05). The addition of 3% and 5% CSFBP-Fermented feed increased the mRNA expression of *ZO-1* and *Occludin* in the jejunum of broiler chickens and reduced the mRNA expression of *IL-6* (*P* < 0.05). The addition of 3% CSFBP-Fermented feed increased the mRNA expression of *Claudin* in the jejunum of broiler chickens and reduced *IL-1β* mRNA expression (*P* < 0.05). Compared to the control group, all experimental groups exhibited decreased mRNA expression of *TNF-α* and INF-γ in the jejunal mucosa of the birds (*P* < 0.05). According to research using high-throughput sequencing of microorganisms' 16S rDNA, and an analysis of α-diversity found that supplementing broilers with 3% CSFBP-Fermented feed decreased the number of bacteria in their cecum (*P* < 0.05). *Bacteroidota* was higher in all groups after supplementation with CSFBP-Fermented feed. At the genus level, after addition with 3% CSFBP-Fermented feed, the abundance of *Bacteroide* and *Prevotellaceae_Ga6A1_group* were higher than the control group (33.36% vs 29.95%, 4.35% vs 2.94%). The abundance of *Rikenellaceae_RC9_gut_group* and *Fusobacterium* were lower than the control group (5.52% vs. 7.17%,0.38% vs. 1.33%). In summary, supplementing the diet with CSFBP-Fermented feed can promote the growth of performance by enhancing intestinal morphology, and barrier function, as well as modulating intestinal inflammatory factors and microbial composition in broilers.

## Introduction

Antibiotics were frequently utilized to preserve health and enhance growth in the chicken industry^1^. Yet, the over-reliance on antibiotics in farming has resulted in large amounts of antibiotics in poultry products, which have become a severe threat to humans and the environment, and most countries have restricted or even prohibited the use of antibiotics as supplements in feed^2,3^. Unfortunately, bans on antibiotics have led to reduced growth performance and increased rates of gut disease^4^. A healthy gut is essential for the function of barriers, microbial community, and nutrient uptake and contributes to enhanced growth and economic efficiency^5,6^. Thus, there is a need to work on new green feed supplements to replace antibiotics for poultry health.

Citri Sarcodactylis Fructus by-products (CSFBP) are by-products of further processing of Citri Sarcodactylis Fructus, such as the production of functional beverages, CSFBP extract, fudge, cosmetics, dried fruit, and jam. However, CSFBP contains a certain amount of nutrients and bioactive components, and these bioactive components can enhance the antioxidant capacity and slow down or inhibit the peroxidation reaction; thus, to some extent, CSFBP has some value for feeding^7,8^. Fermented feeds combine fermentation engineering and enzyme engineering to break down some large molecules into small molecules, which are easily digested and absorbed and produce more active probiotics, various enzymes, metabolites, multiple vitamins, small active peptides, and growth-promoting factors^9,10^. Nowadays, fermented feed is widely used in animal production. Feeding fermented feed enhance broilers' growth capacity, resistance to oxidation and immunity^11,12^. Moreover, the studies revealed that feeding fermented feed can benefit the health of poultry by enhancing the intestinal micro-ecological balance and increasing the digestive capacity and intestinal morphology^13–15^.

Therefore, we supplemented the broiler basal diet with different proportions of CSFBP-Fermented feed for feeding. To study the influence of supplemented CSFBP-fermented feed on growth capacity and biochemical parameters of serum in birds, gut barrier function and intestinal microbiota, to provide data support for the rational use of CSFBP-Fermented feed in poultry breeding, and to offer a scientific basis for broiler healthy farming in the era of antibacterial reduction and replacement.

## Materials and methods

Experimental protocols were approved by the Animal Ethics Committee of the Leshan Academy of Agriculture Sciences (LSNK. No20220701). All the procedures were performed following the Declaration of Basel and relevant policies in China. The study was carried out in compliance with the Animal Research: Reporting of in Vivo Experiments (ARRIVE) guidelines.

### Experimental design and animals

Our trial started on September 24, 2022. For a 42-day feeding study, 1080 47-day-old male cyan-shank partridge birds had separated into four groups (T1–T4). Each group had six replicates (cages) of 45 chickens. Each replicate of 45 broilers was housed in a single cage (120 cm–60 cm–50 cm). Broilers are free to feed and drink throughout the feeding period. Broilers were housed together in a contained way with natural light (about 12.2 h), a temperature range of 18 to 25 °C, and humidity levels of 45% to 55%. Feeding was done twice a day. Group T1 was fed the basal diet, and T2–T4 were supplemented with 1, 3, and 5% CSFBP-Fermented feed in the basal diet, respectively. All chickens in each replicate were weighed at days 21 and 42, after 12 h of starvation, and ADFI, ADG and FCR were measured according to feed consumption.

### Diets

The basal feed formulation and nutrient composition of this trial are present in Table 1, and the CSFBP-Fermented feed formulation and nutrient composition are shown in Table 2. CSFBP in fermented feed comes from Leshan, Sichuan, mainly the trimmings and by-products produced after production and processing. Probiotics used for fermentation include *Lactobacillus Plantarum*, *Saccharomyces cerevisiae* and *Bacillus subtilis*. Enzyme preparations containing xylanase, β-mannanase, β-glucanase and cellulase. They have all been qualified for marketing and the product standard is No. Q/12JX 4450- 2019. Our fermentation process is to inoculate probiotics and enzymes in a glucose solution 24 h before strain activation. Then all raw materials are crushed and passed through 60 mesh sieves, mixed according to the corresponding ratio, put into the fermentation bag containing a one-way breathing valve, inoculated with the activated bacterium solution in advance, and sealed. The fermentation temperature is 36℃, and the fermentation time is 72 h.

**Table 1:** The composition and nutritional level of the basic diet.

Items	Content (%)	Nutritional level^b^	Content (%)
Corn	47.4	Metabolic energy (MJ/kg)	12.83
Cottonseed meal	5	Crude protein	19.5
Chicken powder	4	Crude ash	9.08
Corn bran residue	8	Moisture	11.25
Canola meal	3	Ca	0.73
Corn protein powder	4.9	P	0.37
wheat middling	14	Lysine	0.99
Soybean meal	7	Methionine	0.44
Pork oil	3.35		
CaHPO_4_	1.15		
Stone powder	0.4		
NaCl	0.32		
Lysine	0.48		
Premixes^a^	1		
Total	100		

^a^Premix is provided per kg: V_A_ 8000 IU, V_D_ 1700 IU, V_E_ 13 IU, V_K_ 0.9 mg, VB_1_ 1 mg, VB_2_ 3 mg, VB_6_ 1.3 mg, VB_12_ 0.01 mg, Pantothenic acid 12 mg, Niacin 16 mg, Folic acid 3 mg, Fe 55 mg, Cu 25 mg, Zn 35 mg, Mn 60 mg, Se 0.3 mg, I 0.8 mg.

^b^Nutrient levels are measured except for metabolic energy.

**Table 2:** Composition and nutritional level of CSFBP-fermented feed.

Items	Content (%)	Nutritional level^b^	Content (%)
Corn	10	Metabolic energy (MJ/kg)	8.2
Soybean meal	13.8	Crude protein	24.37
Corn germ meal	3.03	Crude ash	8.88
Corn bran residue	20.6	Crude fiber	2.86
Citri sarcodactylis fructus residues	20	Moisture	33.63
Bran	3	Ca	1.13
Rice husk powder	3	P	0.5
H_2_O	23.72	Lysine	0.87
Stone powder	1.41	Methionine	0.75
Premixes^a^	0.25		
CaHPO_4_	0.46		
NaCl	0.33		
NaHCO_3_	0.1		
Methionine	0.1		
Compound bacteria	0.1		
Enzyme preparation	0.1		
Total	100		

^a^Premix is provided per kg: V_A_ 8000 IU, V_D_ 1700 IU, V_E_ 13 IU, V_K_ 0.9 mg, VB_1_ 1 mg, VB_2_ 3 mg, VB_6_ 1.3 mg, VB_12_ 0.01 mg, Pantothenic acid 12 mg, Niacin 16 mg, Folic acid 3 mg, Fe 55 mg, Cu 25 mg, Zn 35 mg, Mn 60 mg, Se 0.3 mg, I 0.8 mg.

^b^Nutrient levels are measured except for metabolic energy.

### Sample collection

After a 12-h fasting period, one chicken was randomly selected from each repetition, for a total of 24 chickens. First, 5 ml of plasma was drawn from the airfoil sense in a vacuum blood collection tube, left at room temperature for 30 min, and then centrifuged for 10 min at 4 °C at 3000 rpm to extract the serum. The serum was then transferred to a 1.5 ml centrifuge tube and kept at −80 °C in an ultra-low temperature refrigerator with further assessment. The chickens were euthanized using CO_2_ and promptly dissected. An intermediate 2 cm slice of the ileum, jejunum and duodenum were acquired and preserved in 4% paraformaldehyde liquid for paraffin sections after the intestinal contents were removed using pre-cooled saline. Finally, the jejunum of each chicken was collected, and the cecum microorganisms were stored in a −80 refrigerator for further assessment.

### Serum biochemical parameters and enzyme immunoassay

A kit was used to measure the serum's T-AOC, GSH-Px, SOD, CAT and MDA, and the specific methods and processes were carried out by referring to the manual. The enzyme-linked immunoassay (ELISA) analysis was used to evaluate the levels of serum of IgA, IgM and IgG^16^. Briefly, adding 50 ml of standard solution or serum sample to a 96-well plate (loaded with purified chicken IgA, IgG, and IgM antibodies). The second antigen was added to the wells and labeled with horseradish peroxidase. Then the panels were hatched at 37 °C for 60 min. Chromogen solutions were applied and then maintained in the dark for 15 min at 37 °C after the wells had been cleaned five times with wash water. After adding the stop solution, the absorbance was measured at 450 nm using a multipurpose microplate reader.

### Jejunal histomorphology

The paraformaldehyde-fixed jejunal tissue specimens were cut into longitudinal sections, encased with petrolatum, and sliced (5 μm), and then observed morphologically with hematoxylin and eosin staining. The marked segments were initially viewed under low magnification, and the suitable locations were picked for picture capturing under high magnification, all done using a light microscope. With the aid of the picture analysis program Image ProPlus 6.0, villi length and crypt depth were calculated.

### Jejunal mucosa gene expression

Extraction of 50 to 100 mg of total RNA from jejunal mucosa according to manufacturer's instructions (TaKaRa, Japan). By using micro-UV spectroscopy and 1% gel polymeric electrophoresis, RNA quality and concentrations were determined. Following the instructions, the kit (RR047A, TaKaRa) was used to create the first thread of the cDNA. GADPH was utilized as the internal reference gene, and the used primers are displayed in Table 3. Using an overall amount of 20 uL and following the manufacturer's instructions, quantitative real-time PCR (qPCR) was carried out using NovoStart SYBR qPCR Super Mix Plus on a Bio-Rad CFX96. Denaturing for 15 s at 95 °C, heating for 15 s at 56 °C, extending for 30 s at 72 °C, and repeating for 40 cycles were among the thermocycling amplified procedures.

**Table 3:** Primer sequences were used for the real-time PCR analysis.

Genes	Sequence 5′–3′	GenBank number
GAPDH	F: GGAAAGTCATCCCTGAGCTGAAT	NM_204305.1
	R: GGCAGGTCAGGTCAACAACA	
ZO-1	F: AATACCTGACTGTCTTGCAG	XM_015278975.1
	R: TAAAGAAGGCTTTCCCTGAC	
IFN-γ	F: AAAGCCGCACATCAAACACA	NM_205128.1
	R: GCCATCAGGAAGGTTGTTTTTC	
Occludin	F: GCAGATGTCCAGCGGTTACTAC	NM_205128.1
	R: CGAAGAAGCAGATGAGGCAGAG	
Claudin	F: CATACTCCTGGGTCTGGTTGGT	NM_001013611.2
	R: GACAGCCATCCGCATCTTCT	
IL-1β	F: ACTGGGCATCAAGGGCTA	XM_015297469
	R: GGTAGAAGATGAAGCGGGTC	
IL-6	F: GAAATCCCTCCTCGCCAATCT	XM_0152812832
	R: CCTCACGGTCTTCTCCATAAACG	
TNF-α	F: TGTGTATGTGCAGCAACCCGTAGT	NM_204267
	R: GGCATTGCAATTTGGACAGAAGT	

### 16S rDNA sequencing of the cecum microbiota

Microbial DNA was extracted from 200 mg cecal content samples taken from all groups using the MagPure Soil DNA LQ kit. The content and integrity of the DNA were determined using a Thermo Fisher Scientific NanoDrop 2000 spectrophotometer and electrophoresis on agarose gels. The universal primer pairs 343F (5′-TACGGRAGGCAGCAG-3′) and 798R (5′-AGGGTATCTAATCCT-3′) were utilized, as well as TksGflflex DNA polymerase (Takara, R060B). Following that, 30 ml of the reaction mixture was used for PCR amplification of the high variation region V3-V4 in the bacteria's 16S rRNA gene. An Illumina NovaSeq6000 was read using two paired-end read cycles of 250 bases each. OE accomplished the sequencing and profiling of 16S rDNA amplicons (Shanghai, China).

### Statistical analysis

With IBM SPSS Statistics 23, a one-way analysis of variance (ANOVA) was used to assess significance levels. The Tukey multiple range test was used to compare numerous things. *P* < 0.05 was chosen as the cutoff for statistical significance. Data are presented in the table as mean and combined SEM. GraphPad Prism6 was used to create images (GraphPad Software, USA).

## Results

### Growth performance

Table 4 displays how adding CSFBP-fermented feed to the diet affected the growth performance of birds. Supplementation with CSFBP-Fermented feed reduced ADFI (0–21 d, 0–42 d) and reduced FCR at all stages, as well as increased ADG (0–21 d, 0–42 d) in broilers (*p* < 0.05). Throughout the trial period, the 3% addition was highest for ADG and lowest for ADFI and FCR compared to all test groups.

**Table 4:** The effects of CSFBP-fermented feed supplementation in diets on the growth performance of birds.

Items	T1	T2	T3	T4	SEM	P-value
Starter phase (days 0–21)						
ADFI (g/day)	137.83^a^	132.12^b^	131.43^b^	131.45^b^	0.73	< 0.05
ADG (g/day)	29.54^c^	35.74^b^	41.41^a^	41.93^a^	1.30	< 0.05
FCR (g/g)	4.74^a^	3.73^b^	3.21^bc^	3.15^c^	0.16	< 0.05
Grower phase (days 22–42)						
ADFI (g/day)	175.57^a^	172.35^ab^	166.69^b^	170.09^ab^	1.16	< 0.05
ADG (g/day)	39.26	45.95	47.27	41.80	1.36	0.130
FCR (g/g)	4.35^a^	3.62^b^	3.42^b^	3.94^ab^	0.13	0.048
Whole phase (days 0–42)						
ADFI (g/day)	156.70^a^	152.23^b^	149.06^b^	150.77^b^	0.81	< 0.05
ADG (g/day)	34.40^c^	40.85^b^	44.34^a^	41.87^ab^	0.90	< 0.05
FCR (g/g)	4.48^a^	3.63^b^	3.28^c^	3.51^bc^	0.11	< 0.05

*T1* control supplied with the basal diets, *T2* 1% CSFBP-fermented feed replacement group, *T3* 3% CSFBP-fermented feed replacement group, *T4* 5% CSFBP-fermented feed replacement group, *FCR* feed conversion rate, *ADFI* average daily feed intake, *ADG* average daily gain.

^a–^^c^Significant difference in the mean of different letters in a row (n = 6, *P* < 0.05).

### Serum immune and antioxidant function

As shown in Table 5, adding CSFBP-Fermented feed in the basal diet had no significant effects on TP, IgA, IgM, IgG, and SOD of broiler serum (*p* > 0.05). Supplementation with 3% and 5% CSFBP-Fermented feed significantly reduced GSH-Px in broiler serum (*p* < 0.05). The addition of 1% and 3% CSFBP-Fermented feed significantly increased CAT in broiler serum (*p* < 0.05). The MDA content in broilers supplemented with CSFBP-Fermented feed were significantly smaller than the control group (*p* < 0.05).

**Table 5:** The effects of CSFBP-fermented feed supplementation in diets on serum immune and antioxidant function of broilers.

Items	T1	T2	T3	T4	SEM	P-value
TP(g/L)	35.79	43.72	36.35	40.03	2.18	0.573
IgA(g/L)	5.94	5.85	5.96	7.43	0.36	0.351
IgM (g/L)	8.44	9.06	8.39	10.52	0.35	0.106
IgG (g/L)	29.93	24.03	33.26	27.41	1.54	0.183
SOD (U/mL)	16.70	17.05	17.19	16.38	0.61	0.971
GSH-Px(U/mL)	1312.50^a^	1312.50^a^	1056.25^b^	937.50^b^	48.28	< 0.05
T-AOC (U/μL)	1.08a^b^	0.97b^c^	1.28^a^	0.78^c^	0.06	< 0.05
CAT (U/mL)	4.21^b^	9.28^a^	9.40^a^	7.29^ab^	0.67	< 0.05
MDA (mol/mL)	5.63^a^	1.85^b^	2.21^b^	1.35^b^	0.39	< 0.05

*TP* total protein, *Ig* immunoglobulin, *CAT* catalase, *T-AOC* total antioxidant capacity, *SOD* superoxide dismutase, *GSH-Px* glutathione peroxidase, *MDA* malondialdehyde.

^a,b^Significant difference in the mean of different letters in a row (n = 6, *P* < 0.05).

### Small intestinal histomorphology

**Figure 1:**
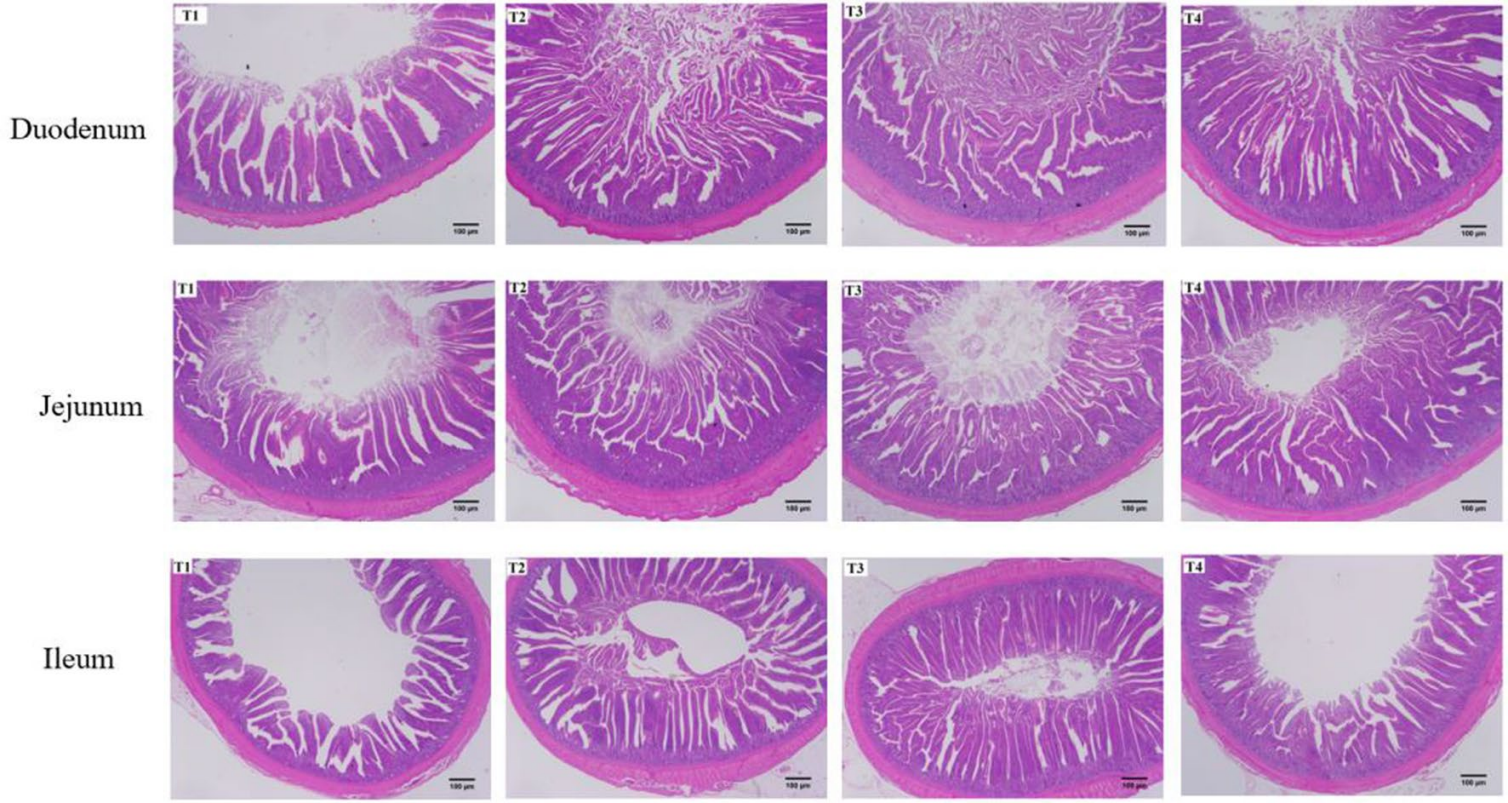
Morphological structure of the intestine (duodenum, jejunum, and ileum) of broiler chickens on day 42.

The microscopic photographs of the small intestine morphology are shown in Fig. 1. The height of the intestinal villi is shown in Table 6. It can be observed that the addition of CSFBP-Fermented feed enhanced the height of the villi in the small intestine broiler. CSFBP-Fermented feed significantly increased VH in broilers’ duodenum, jejunum, and ileum in birds (*P* < 0.05). Supplementation with 3% CSFBP-Fermented feed increased CD in the duodenum and decreased CD in the jejunum of broilers (*P* < 0.05). VH/CD in broiler duodenum, jejunum and ileum was elevated by supplementation with CSFBP-Fermented feed.

**Table 6:** Effects of CSFBP-Fermented feed supplementation on intestinal morphology of broilers.

Items	T1	T2	T3	T4	SEM	P-value
Duodenum						
VH (µm)	643.36^c^	965.03^b^	1101.62^a^	1105.69^a^	40.71	< 0.05
CD (µm)	107.14^b^	105.53^b^	141.40^a^	116.20^b^	3.64	< 0.05
VH/CD	6.03^b^	9.18^a^	7.85^b^	9.60^a^	0.33	< 0.05
Jejunum						
VH (µm)	726.01^c^	1113.69^a^	992.31^b^	1079.53^a^	32.27	< 0.0*5*
CD (µm)	159.23^a^	166.34^a^	113.45^b^	110.95^b^	5.66	0.130
VH/CD	4.59^d^	6.72^c^	8.79^a^	9.76^b^	0.43	0.048
Ileum						
VH (µm)	527.31^c^	652.99^b^	733.07^a^	653.08^b^	15.60	< 0.05
CD (µm)	90.11^c^	97.14^a^	92.22^bc^	109.07^b^	1.86	< 0.05
VH/CD	5.86^c^	6.74^b^	8.00^a^	6.00^c^	0.20	< 0.05

*VH* villous height, *CD* crypt depth, *VH/CD* villous height/crypt depth.

^a–^^d^Significant difference in the mean of different letters in a row (n = 6, *P* < 0.05).

### The expression levels of mRNA for tight junction proteins and immune-regulatory genes

**Figure 2:**
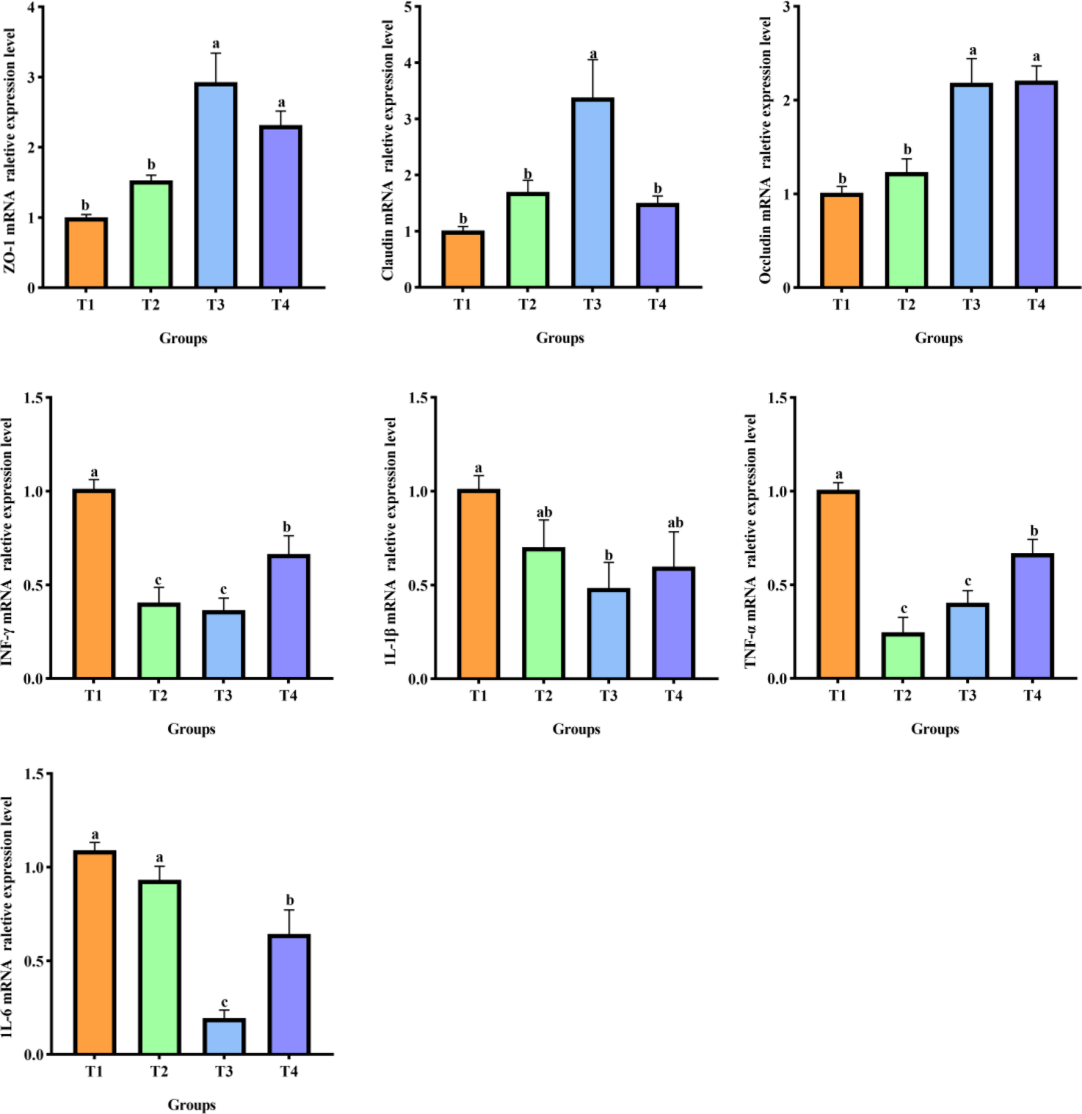
The expression levels of mRNA of immunoregulatory genes and tight junction proteins in birds jejunal mucosa. Different letters indicate significant differences (n = 6, *P* < 0.05); values are presented as mean ± SEM (n = 6); *INF-γ* interferon-γ, *TNF-α* tumor necrosis factor-α, *IL-1β* interleukin-1β, *IL-6* interleukin-6, *ZO-1* zonula occluden-1.

The expression of mRNA of immune regulatory and genes tight junction proteins in birds jejunal mucosa were presented in Fig. 2. Supplementation of the basal diet with 3 and 5% CSFBP-Fermented feed enhanced *ZO-1* and *Occludin* mRNA expression in the broiler chicken jejunal mucosa (*P* < 0.05). Supplementation with 3% CSFBP-Fermented feed significantly increased the mRNA expression of *Claudin* in broiler jejunal mucosa (*P* < 0.05). Supplementing feed with 3% CSFBP-Fermented feed decreased *IL-1β* mRNA expression in the jejunum, and supplementation with 3 and 5% CSFBP-Fermented feed significantly reduced the mRNA expression of *IL-6* in the jejunum of broilers (*P* < 0.05). Compared with the control group, all experimental groups showed downregulation of *TNF-α* and *INF-γ* mRNA expression in the jejunal mucosa of birds (*P* < 0.05).

### Cecal microbiota analysis by 16S rDNA

**Figure 3:**
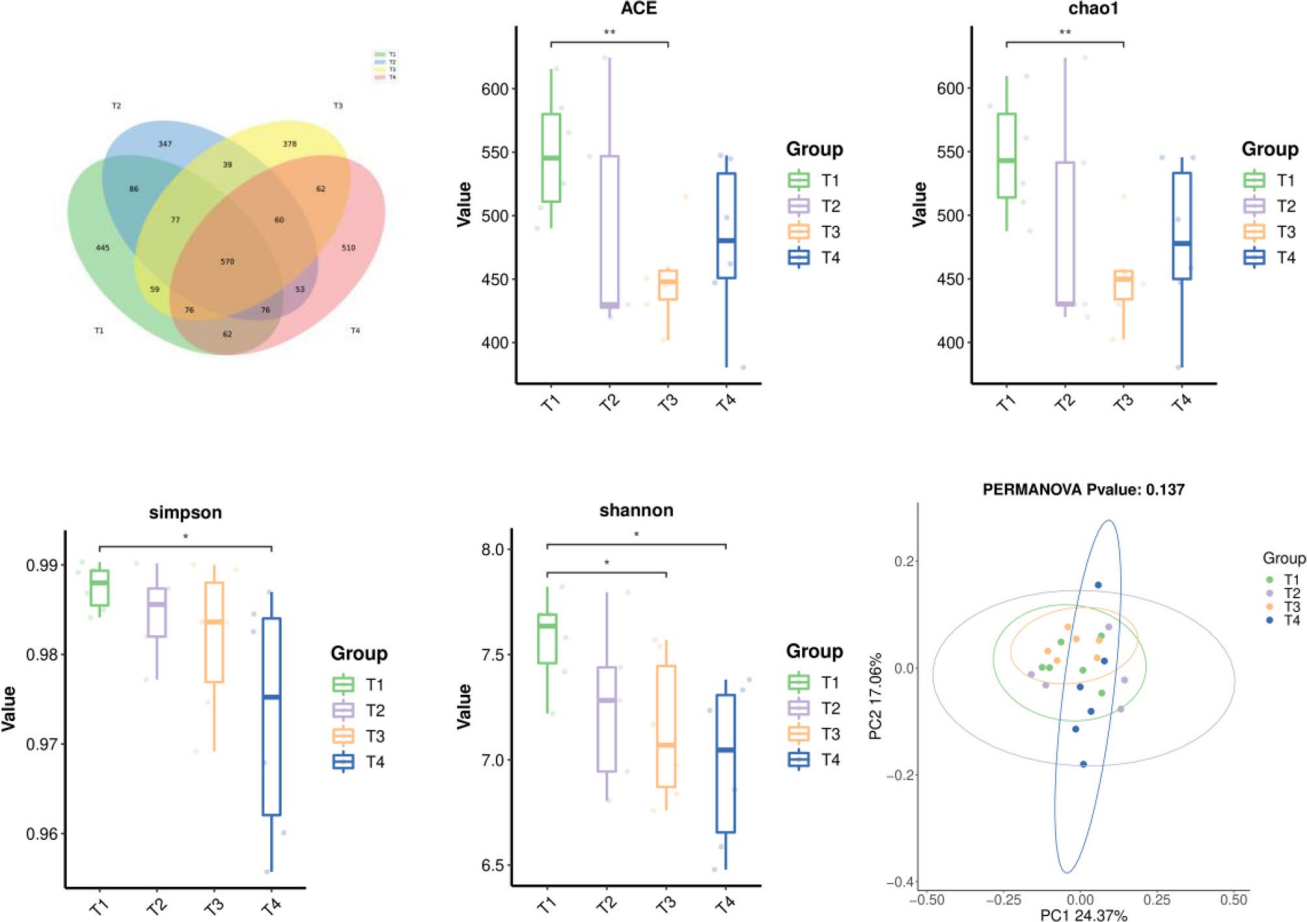
Effects of dietary supplementation with CSFBP-Fermented feed on the cecum microbial Venn diagram, α-diversity and β-diversity of broilers. Principal coordinate analysis (PCoA) based on weighted UniFrac distance calculated from OTU abundance matrix; asterisk means the significant difference between the two groups (*p < 0.05, **p < 0.01).

The cecal samples taken from broilers contained several 2900 OTUs. 570 OTUs were shared by the four groups, whereas 445, 347, 378, and 510, respectively, OTUs were unique to the T1, T2, T3, and T4 groups (Fig. 3A). We used the abundance and diversity indices of Simpson, Shannon, ACE and Chao1 to estimate the bacterial alpha-diversity of the cecum microbiota and the results are shown in Fig. 3. Compared with the control group, supplementation with 3% CSFBP-Fermented feed significantly reduced the cecum microbial Chao1, ACE and Shannon indices in broiler chickens (*P* < 0.01). The addition of 5% CSFBP-Fermented feed significantly decreased the Shannon and Simpson indices in broilers (*P* < 0.05). The total microbial profiles of the four groups were compared using the beta diversity analysis, as shown in Fig. 3. To offer a comprehensive understanding of the microbiota using the weighted UniFrac distance measure, PCoA analysis was carried out. The PCOA results revealed that there was no real difference between the four groups of microbial samples (*P* > 0.05).

**Figure 4:**
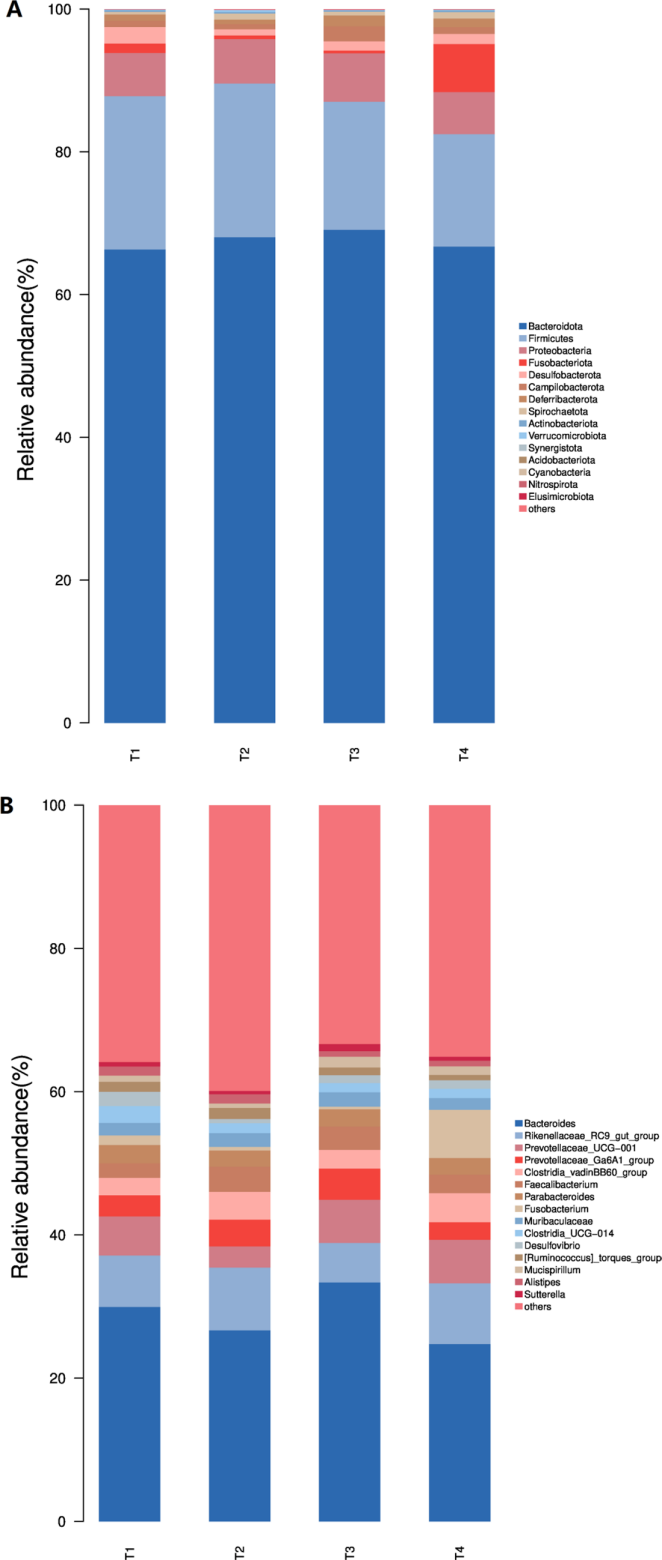
The composition of the microbiota and characterization of the distinct species in the cecum of birds. (**A,B**) Bacterial community composition at the phylum and genus level, respectively.

To evaluate the differences caused by the CSFBP-Fermented feed in the cecum microbiota, we analyzed the classification and composition of the cecum microbiota at both the phylum and genus levels (Fig. 4). At the phylum level, we identified 15 different species of bacteria. More than 80% of the sequences in all test groups were of the two most common bacterial phyla, *Bacteroidota* and *Firmicutes* (Fig. 4A). *Bacteroidota* was higher in all groups after feeding CSFBP-Fermented feed than in the control group (*P* > 0.05). The *Firmicutes* were lower in the cecum of broilers supplemented with 3% and 5% CSFBP-Fermented feed than in the control group (17.93%, 15.75% vs 21.48%, *P* > 0.05). There was a decrease in *Desulfobacterota* in each group after feeding CSFBP-Fermented feed compared with the control group (2.31%,0.87%,1.30%,1.42%, *P* > 0.05). *Campilobacterota* and *Deferribacterota* were highest in the cecum of broiler chickens supplemented with 3% CSFBP-Fermented feed (2.11% and 1.52%).

We discovered the top 15 most prevalent genera at the genus level (Fig. 4B). The *Bacteroides*, *Rikenellaceae RC9_gut_group*, *Prevotellaceae_UCG-001*, *Prevotellaceae_Ga6A1_group*, *Clostridia_vadinBB60_group*, and *Faecalibacterium* species dominated the four groups. Supplementation of CSFBP-Fermented feed in broiler diets enhanced the abundance of *Muribaculaceae* and *Mucispirillum* and decreased the abundance of *Clostridia_UCG-014 *(*P* > 0.05). Supplementation with CSFBP-Fermented feed decreased the abundance of *Desulfovibrio* (*P* < 0.05). Supplementation with 3% CSFBP-Fermented feed improved the abundance of *Bacteroides* and *Prevotellaceae_Ga6A1_group* (33.36% vs 29.95%, 4.35% vs 2.94%) and decreased the abundance of *Rikenellaceae_ RC9_gut_group* and *Fusobacterium* (5.52% vs. 7.17%,0.38% vs. 1.33%).

## Discussion

The fermented feed is easier to be digested and absorbed by animals. The anti-nutritional factors in the spread can be removed to enhance the nutritional quality palatability and digestibility of the feed, thus improving the growth performance of poultry^17,18^. In the present study, our data suggest that feeding CSFBP-Fermented feed can increase ADG and decrease ADFI and FCR in broilers. It was demonstrated that providing fermented feed can promote growth performance by facilitating the digestion and absorption of feed in poultry. However, it is interesting to note that our results showed that feeding CSFBP-Fermented feed reduced ADFI in broilers, which is contrary to some studies. The reason may be that the palatability of CSFBP-Fermented feed affects the intake of broilers. Still, the increase in ADG of broilers is another good result, which is that CSFBP-Fermented feed increases the digestion and absorption in broilers, thus enhancing the utilization of feed and promoting growth performance. In conclusion, the specific reasons need further verification.

IgA, IgG, and IgM are important immune molecules in the body and are involved in various immune responses. According to reports, adding supplements to diets with Bio-Fermented Malic Acid, fermented feed, and Fermented Fava Bean By-Products can increase serum IgA, IgG, and IgM levels in poultry^11,19–21^. Our study found a slight increasing trend in the levels of IgA, IgG, and IgM in broiler chickens when adding 3% CSFBP-Fermented feed, but there were no significant differences compared to the control group. This may be explained by the combined effect of probiotics in the fermented feed and active substances in the Citri Sarcodactylis Fructus, which promote the synthesis of vitamins, amino acids, and other substances in the intestine of animals and serve as antigens and nutrients to stimulate and encourage the development of immune organs^22–24^. In animals, the production of free radicals is in balance with the antioxidant system in the body, and this balance is crucial in sustaining the health of the animals^25^. Antioxidant enzymes play a vital role in the body's natural defense against oxidative degradation. Antioxidant enzymes mainly include SOD, GSH-Px and CAT, which can cooperate and scavenge free radicals and reactive oxygen species by preventing peroxide production, avoiding lipid peroxidation, and eliminating metal ions to achieve the purpose of antioxidation^26–28^. In addition, MDA is also an important indicator to evaluate the body's antioxidant function and cellular oxidative stress. It is a lipid peroxidation product, and its concentration reflects the extent of internal oxidative damage^29^. It has been found that feeding fermented pine needles (Pinus ponderosa) improved the activity of serum and liver SOD and GSH-Px and decreased the concentration of MDA in broiler chickens^30^. Feeding fermented ginkgo improved broiler intestinal health and boosted antioxidant capacity by raising SOD and GSH-Px activity and reducing MDA contents in the jejunum and ileum^31^. In line with our findings, we discovered that providing CSFBP-Fermented feed boosted SOD and CAT activity while lowering MDA levels in broiler serum. These findings imply that adding CSFBP-Fermented feed to broiler diets is useful for preventing lipid peroxidation. This could result from bacteria and functional ingredients (polysaccharides, phenols, and flavonoids), which could also be an effective mechanism to lessen the load on the enzymatic and non-enzymatic antioxidant systems to lower cellular antioxidant consumption.

Animals primarily digest and absorb nutrients through the gut, and the shape of the intestine can provide insight into the animal's intestinal health and capacity for nutrient absorption^32^. VH and CD are significant measures of digestive and absorption function in the intestine. An increase in VH can increase the contact area of nutrients with the intestine and promote nutrient absorption. At the same time, a decrease in CD indicates a shorter maturation cycle of intestinal epithelial cells, increased secretion and enhanced digestive capacity. The VH/CD of both can also reflect the development of the intestinal tract and digestive and absorption ability to a certain extent^33,34^. Fermented feed was reported to raise chicks' VH and VH/CD of the gut (duodenum, jejunum, and ileum)^35^. Similarly, many studies have shown that feeding fermented feeds can facilitate the digestion and absorption in poultry by improving intestinal morphology^36–38^. Our study found that supplementation of CSFBP-Fermented feed reduced CD in broiler chickens. We speculate that this may be due to the ability of CSFBP-Fermented feed to regulate the balance of intestinal microbiota, provide beneficial metabolites, and improve intestinal barrier function, thereby lowering the depth of intestinal crypts and maintaining intestinal health. Our study found that supplementation with CSFBP-Fermented feed can increase the gut morphology of broiler chickens by increasing intestinal VH and reducing CD, thus promoting the body's ability to digest and absorb nutrients and improving growth performance.

The important protein components in tight junctions are claudin, occludin, and *ZO-1*, and tight junctions are cellular gaps between neighboring epithelial cells^39^. The function of Claudin is mainly to regulate the permeability of the barrier structure; *Ocludin* can maintain tight junction stability and small intestinal permeability; *ZO-1* can maintain epithelial cell polarity and gut barrier permeability^40,41^. Our study found that supplementation with CSFBP-Fermented feed increased the relative mRNA expression of *Claudin*, *Occludin* and *ZO-1* in broiler jejunum. Consistent with this study, supplementation of the basal diet with Fava Bean By-Products, Laetiporus sulphureu, and Fermented Soybean Meal increased intestinal tight junction protein in poultry^21,36,42^. Supplementation with CSFBP-Fermented feed to the diet can enhance the tight junctions of gut epithelial cells by promoting the expression of intestinal tight junction protein-related genes, which has a specific effect on the maintenance of the mechanical barrier function of the broiler intestine. The *IL* family has a key function in maintaining the integrity of the gut mucosa, controlling gut immunology, and modulating several pathways involved in apoptosis or inflammation. *IL-1* and *IL-6* are among the most significant regulators of inflammation^43,44^. *TNF-α*, a crucial pro-inflammatory factor, stimulates macrophages and controls inflammation and death of cells. *IFN-γ* linked to infection, and having excessive amounts of it can cause autoimmune diseases^45,46^. Our results show that CSFBP-Fermented feed can exert anti-inflammatory effects by inhibiting the overexpression of *IL-1β*, *IL-6*, *TNF-α* and *IFN-γ*. This is consistent with previous studies that feeding fermented feed can regulate the mRNA expression levels of inflammatory factors in poultry^42,47^.

The cecum of broiler chickens is an essential region for fermenting and digestion, which is critical to poultry health and immunity, and is the subject of a gut microbial study^48^. Many factors affect the microbiology of the cecum, including feed composition, breeding environment, age, genetics and other factors^49,50^. The gut microbiota controls a variety of host metabolic processes, influencing immune response, protecting gut wall integrity, and secreting helpful enzymes and metabolites^51,52^. The OTU data show the number of bacterial communities. The OTU data represent the number of bacterial communities; the alpha diversity index is frequently used to describe the variety and abundance of bacterial diversity. The Shannon and Simpson indices represent the microbial diversity in the samples^53,54^. Surprisingly, the addition of CSFBP-Fermented feed lowered the Simpson ACE, Shannon and Chao1 microorganism indices in the broiler cecum, indicating a drop in microbiota diversity and richness in the broiler cecum. This is related to our classification in the phylum and genus of intestinal microorganisms. We discovered that *Bacteroidota* and *Firmicutes* make up most of the microbes in the cecum microbiota of birds. These organisms contribute to further than 80% of the abundances of the gut microbiome and are crucial for sustaining gut balance^55^. The abundance of *Bacteroidota* in the broiler cecum was higher in the CSFBP-Fermented feed than in the control group, and *Bacteroides* species can use complex polysaccharides as a source of carbon and energy for fermentation and metabolism, releasing fermentation end-products that also provide nutrients and other beneficial properties for the host^56^. Therefore, we hypothesise that the higher abundance of *Bacteroidota* after supplementation with CSFBP-Fermented feed may help broilers adapt to the ration and improve the digestibility of nutrients. It was found that the bacterial community associated with body weight, the body weight of geese was highly correlated with members of the *Bacteroidaceae*, that is, members of the *Bacteroidaceae* were significantly enriched in geese with high body weight^53^. Similarly, this research found that broilers fed CSFBP-Fermented feed had higher body weight and ADG, suggesting that *Bacteroidetes* bacteria may be positively associated with broiler growth performance. Supplementation with CSFBP-Fermented feed reduced the abundance of harmful bacteria *Desulfobacterota* in the broiler cecum^57^ and increased the abundance of some beneficial bacteria such as *Campilobacterota* and *Deferribacterota* in the broiler cecum^58,59^.

Each group's predominant flora at the genus level is *Bacteroides* and *Rikenellaceae_RC9_gut_group*, similar to Zhu's study^54^, but inconsistent with Wang's results^60^. The differences in results may be related to chicken breed, diet, age and rearing environment^54^. Supplementation of 3% CSFBP-Fermented feed increased the abundance of *Muribaculaceae* and *Prevotellaceae_Ga6A1_group* in broiler chickens. A study finds potential benefits of *Muribaculaceae* in reducing inflammation, inhibiting harmful bacteria and promoting anti-cancer immunity^61^. Our data showed that feeding CSFBP-Fermented feed significantly decreased the abundance of *Desulfovibrio* and *Fusobacterium*. *Desulfovibrio* genus causes the host to mitigate dietary sulfites and sulfates, aand sulfated mucopolysaccharides, leading to the production of a cytotoxic compound hydrogen sulfide^62^. According to studies, those with ulcerative colitis had a higher prevalence of *Desulfovibrio* and a more pronounced inflammatory response^63^. In broilers treated with CSFBP-Fermented feed, we discovered that the species richness of *Desulfovibrio* was decreased, indicating that the CSFBP-Fermented feed may exercise its anti-inflammatory action by lowering the quantity of sulfate-reducing *Desulfovibrio*^64^. These findings demonstrate that adding CSFBP-Fermented feed to a diet can enhance the number of some helpful bacteria while reducing the prevalence of some harmful bacteria, benefiting the intestinal health of broilers. The reason for the decrease in microbial diversity and abundance after supplementation with CSFBP-Fermented feed may be that supplementation with CSFBP-Fermented feed promoted the colonization of thcolonisation by some probiotic bacteria and inhibiting the growth of some pathogenic bacteria, thus decreasing microbial abundance. However, these causal relationships need to be further investigated.

In conclusion, our research indicates that supplementation of CSFBP-fermented feed is beneficial for broiler chickens. In the current context of advocating for the prohibition or reduction of antibiotic use in animal production, we incorporated Citri Sarcodactylis Fructus by-products as part of the fermented feed formulation, which differs from conventional fermented feed. Specifically, CSFBP-fermented feed includes certain advantageous functions derived from Citri Sarcodactylis Fructus, such as antibacterial, anti-inflammatory, antiviral, and immunomodulatory properties. This demonstrates that CSFBP-fermented feed exhibits certain advantages over regular fermented feed. However, due to the complexity of CSFBP-fermented feed itself, which includes components like Citri Sarcodactylis Fructus, probiotics, enzyme preparations, and metabolic by-products, it is difficult to determine which specific component promotes the growth of broiler chickens. In future studies, researchers should conduct more in-depth research to unveil whether it is a single component or the synergistic effect of multiple components that promotes the growth of broiler chickens.

## Conclusions

The current findings suggest that supplementing CSFBP-fermented feed can enhance growth performance by improving gut morphology, and barrier function, modulating the intestinal inflammatory response and intestinal microbial composition in broilers. Combining all the data analysed, a 3% supplementation level is more advantageous.

## Data Availability

The sequencing data from this study can be found at: https://www.ncbi.nlm.nih.gov/sra, and the Accession number is PRJNA940200.
